# Transcriptome Analysis and Its Application in Identifying Genes Associated with Fruiting Body Development in Basidiomycete *Hypsizygus marmoreus*

**DOI:** 10.1371/journal.pone.0123025

**Published:** 2015-04-02

**Authors:** Jinjing Zhang, Ang Ren, Hui Chen, Mingwen Zhao, Liang Shi, Mingjie Chen, Hong Wang, Zhiyong Feng

**Affiliations:** 1 College of Life Science, Nanjing Agricultural University, Key Laboratory of Microbiological Engineering of Agricultural Environment, Ministry of Agriculture, Nanjing, Jiangsu, China; 2 National Research Center for Edible Fungi Biotechnology and Engineering, Key Laboratory of Applied Mycological Resources and Utilization, Ministry of Agriculture, the People’s Republic of China, Shanghai Key Laboratory of Agricultural Genetics and Breeding, Institute of Edible Fungi, Shanghai Academy of Agricultural Sciences, Shanghai, China; Ruhr-University Bochum, GERMANY

## Abstract

To elucidate the mechanisms of fruit body development in *H*. *marmoreus*, a total of 43609521 high-quality RNA-seq reads were obtained from four developmental stages, including the mycelial knot (H-M), mycelial pigmentation (H-V), primordium (H-P) and fruiting body (H-F) stages. These reads were assembled to obtain 40568 unigenes with an average length of 1074 bp. A total of 26800 (66.06%) unigenes were annotated and analyzed with the Kyoto Encyclopedia of Genes and Genomes (KEGG), Gene Ontology (GO), and Eukaryotic Orthologous Group (KOG) databases. Differentially expressed genes (DEGs) from the four transcriptomes were analyzed. The KEGG enrichment analysis revealed that the mycelium pigmentation stage was associated with the MAPK, cAMP, and blue light signal transduction pathways. In addition, expression of the two-component system members changed with the transition from H-M to H-V, suggesting that light affected the expression of genes related to fruit body initiation in *H*. *marmoreus*. During the transition from H-V to H-P, stress signals associated with MAPK, cAMP and ROS signals might be the most important inducers. Our data suggested that nitrogen starvation might be one of the most important factors in promoting fruit body maturation, and nitrogen metabolism and mTOR signaling pathway were associated with this process. In addition, 30 genes of interest were analyzed by quantitative real-time PCR to verify their expression profiles at the four developmental stages. This study advances our understanding of the molecular mechanism of fruiting body development in *H*. *marmoreus* by identifying a wealth of new genes that may play important roles in mushroom morphogenesis.

## Introduction

The process of mushroom formation has been a popular topic in mycological research. The aim of mushroom formation research is to understand the molecular mechanisms underlying fruiting body (F-B) initiation and development in mushroom-forming basidiomycetes. Many studies of mushroom development have focused on the model species *Coprinopsis cinerea* and *Schizophyllum commune* [1] However, only a relatively small number of studies have been performed on non-model mushroom development, including in the commercially important *Agaricus bisporus*, *Flammulina velutipes*, and *Boletus edulis* [2] species. The fruiting process is one of the most complex, yet rapid, developmental events in the life cycle of mushrooms. Fruiting is regulated by cellular processes, and genetic, physiological and environmental factors are all involved in normal fruiting [3]. Many edible mushrooms cannot be commercially cultivated due to the poor understanding of F-B development. For most commercially important mushroom species, limited research has been conducted on these processes primarily because suitable analysis tools are not available [4–5]. Therefore, further study will aid in the understanding of the fruiting process of non-model mushrooms and consequently have a commercial impact.

In mushrooms, the fruiting body process can be divided into four main developmental stages: the hyphal knot, initial primordium, primordium and fruiting body [1]. Several genes have been cloned and studied at different stages of mushroom morphogenesis in certain model species. It has been found that some genes function differentially at different developmental stages, such as Noxs [6], *dst* [7–8], *Ubc2* [9] and *eln* [10–11]. In addition to these functional genes, there are likely many transcription factors involved in sexual development; however, only a limited number have been identified, such as WC-1 [12], *FlbB* [13] and *Pro1* [14]. These transcription factors have important functions in regulating the development of fungi. Moreover, some studies have reported on the signaling pathways that are involved in regulating sexual development, such as the MAPK [15], and cAMP/PKA signaling pathways [16–17].

*Hypsizygus marmoreus* mushrooms are in the Agaricales Tricholomataceae order and are widely cultivated and sold in East Asia. The fruiting process of *H*. *marmoreus* can be divided into four stages: mycelial knot (H-M), mycelial pigmentation (H-V), primordium (H-P) and fruiting body (H-F). Recent research has shown that *H*. *marmoreus* is a useful model for studying the developmental process of non-model mushrooms [18–19] given that commercial mushroom growing does not involve a great deal of knowledge of F-B. In addition, molecular genetics studies have also been conducted, resulting in the cloning and characterization of *H*. *marmoreus* genes [20]. Zhang et al. [21] constructed an efficient Agrobacterium-mediated transformation method for *H*. *marmoreus* that can be used to study gene functions during the developmental process.

In recent years, Illumina sequencing techniques have facilitated research in the life sciences and have dramatically improved the efficiency of gene discovery [22–23]. In *C*. *cinerea*, the transcriptomes from the mycelium to the initial primordium stages has been studied. The results showed that the transcriptomic changes were the most significant at the late stage of fruit body formation during the development from an undifferentiated structure to a well-organized multi-tissue fruit body structure [22]. The transcriptomes of the mycelium and fruit body were also studied in *A*. *bisporus*, *Amanita exitialis* and *Agrocybe aegerita*, and a great deal of information on the transition from mycelium to fruit body was obtained [24–26]. However, for non-model mushrooms, such as *H*. *marmoreus*, information is lacking regarding the developmental mechanisms underlying the transitions from (i) the mycelial knot to the mycelial pigmentation stage, (ii) the mycelial pigmentation to the primordium stage, and (iii) the primordium to the fruit body stage. This lack of understanding hinders the generation of a more comprehensive picture of the fruiting process of mushrooms.

To better understand the molecular mechanism of mushroom fruit body development, the transcriptomes of four developmental stages of *H*. *marmoreus* were analyzed in this study. RNA-seq was performed using Illumina technology, which produced comprehensive information on gene expression at the transcriptional level. This transcriptomic information could facilitate our understanding of the genetic and molecular mechanisms of fruiting body development in *H*. *marmoreus*. Furthermore, the results for *H*. *marmoreus* could be used as important resources by which to investigate the H-F pathways in other non-model basidiomycetes.

## Materials and Methods

### Sample preparation

*H*. *marmoreus* samples were obtained from the China General Microbiological Culture Collection Center (Beijing) (no. CGMCC5.01974). The liquid culture of this strain was transferred to basswood medium (sterilized at 121°C and 0.1 MPa for 2.5 h) and cultured in the dark at 25°C. After the mycelia grew in a spawn-running process (60–70 days) to allow mycelial maturation, the mycelium was sampled. The bottles were then placed under continuous light at 14–16°C. The light intensity was kept at 50–100 lux during the first 5 days and subsequently increased to 200–1000 lux at the 6^th^ day. On approximately the 7^th^ day, the white mycelia turned brown, and the primordium appeared near the 10^th^ day; the brown mycelia and primordia were then collected. After the fruit bodies maturated, the fruit bodies with the stipe and pileus were collected on the 22^nd^ day. Samples from three randomly selected trays at the four developmental stages were frozen at -80°C for RNA extraction.

### RNA extraction, cDNA library construction and Illumina sequencing

To obtain an overview of the gene expression profiles of *H*. *marmoreus* during four developmental stages, cDNA samples were prepared from the H-M, H-V, H-P and H-F stages of *H*. *marmoreus*. The transcriptomes of these stages were sequenced separately using the Illumina HiSeqTM 2000 sequencing platform. Total RNA from the four samples of *H*. *marmoreus* were extracted using TRIzol reagent (Takara, Japan), according to the manufacturer’s protocol. To avoid the interference of proteins and polysaccharides, the RNA quantity and quality were evaluated using a ND-2000 spectrophotometer (NanoDrop Technologies) and a 2100 Bioanalyzer (Agilent Technologies, Santa Clara, CA). RNA-seq transcriptome libraries were prepared using the TruSeq RNA sample preparation Kit from Illumina (San Diego, CA). Briefly, messenger RNA was isolated using oligo(dT) beads and fragmented in fragmentation buffer. The cDNA synthesis, end repair, A-base addition and ligation of the Illumina-indexed adaptors were performed according to Illumina’s protocol. The libraries were then size-selected for cDNA target fragments of 200–300 bp on 2% Low Range Ultra Agarose gel. This step was followed by PCR amplification (15 cycles) using Phusion DNA polymerase (NEB). After quantification using a TBS-380 Mini-Fluorometer, paired-end libraries were sequenced with an Illumina HiSeq 2000 sequences (2 × 100 bp read length). The raw data from the four samples have been submitted separately to the National Center for Biotechnology Information (NCBI) under the accession number SRP040755, and the Transcriptome Shotgun Assembly project has been deposited at DDBJ/EMBL/GenBank under the accession GBCL00000000. The version described in this study is the first version, GBCL01000000.

### De novo assembly and Annotation

The raw paired-end reads were trimmed of the adaptor sequences for quality control using SeqPrep (https://github.com/jstjohn/SeqPrep) and Sickle (https://github.com/najoshi/sickle), with default parameters. Then, clean data of the four samples were used to perform RNA de novo assembly with Trinity (http://trinityrnaseq.sourceforge.net/) [27]. All of the assembled transcripts were defined as unigenes. The ESTScan program was used to analyze the open reading frame (ORF) of the unigenes. All of the unigenes (S1 Dataset) were predicted and annotated using local BLASTX programs against the NCBI nr/nt, SwissProt, STRING [28] and Cluster of Orthologous Groups (COG) databases (10^-5^ E-value cutoff). BLAST2GO (http://www.blast2go.com/b2ghome) [29] was used to obtain GO annotations of unique assembled transcripts for defining biological processes, molecular functions and cellular components. The metabolic pathway analysis was performed using the Kyoto Encyclopedia of Genes and Genomes (KEGG, http://www.genome.jp/kegg/) [30].

### Analysis of differentially expressed genes (DGEs)

Differentially expressed genes (DEGs) between two libraries were identified by the programs RSEM (http://deweylab.biostat.wisc.edu/rsem/) [31] and edgeR (Empirical analysis of Digital Gene Expression in R, http://www.bioconductor.org/packages/elease/bioc/tml/edgeR.html) package [32]. RSEM was used firstly to calculate the mapping read counts to every assembled unigenes and the expression level of each unigene according to the fragments per kilobase of exon per million mapped reads (FPKM) [33]. Then, edgeR was used to trim the unigenes counts obtained by RSEM and analyses differential expression gene. The p-value in multiple tests was determined by the value for the false discovery rate (FDR). We used ‘FDR < = 0.05 and | log2FC | > = 2’ as the threshold to judge the significance of gene expression differences. In addition, functional-enrichment analysis, including GO and KEGG analysis, were performed to identify significantly enriched DEGs in GO terms and metabolic pathways compared with the whole-transcriptome background. For this analysis, a Bonferroni-corrected P-value ≤0.05 was used. GO functional enrichment and KEGG pathway analyses were carried out using Goatools (https://github.com/tanghaibao/Goatools) and KOBAS (http://kobas.cbi.pku.edu.cn/home.do) [34].

### Quantitative real-time PCR (qRT-PCR) validation

Approximately 2 μg of total RNA from the four stages were reverse-transcribed by M-MLV reverse transcriptase (Takara) using oligo (dT) as the primer. The unigenes of interest were subjected to quantitative real-time PCR (qRT-PCR) analysis. The primers and accession numbers of these genes and the internal reference gene (18S ribosomal RNA) are listed in S1 Table. The amplifications were performed using 0.4 μl (10 μM) of specific primers, 10 μl of SYBR qPCR Mix (Takara), 0.4 μl Rox (Takara) and 2.0 μl cDNA in a final volume of 20 μl. The cycling parameters were 95°C for 5 min followed by 30 cycles of 95°C for 5 s, 60°C for 15 s and 72°C for 20 s. Three independent biological replicates were performed for each gene tested in real time PCR reactions. The relative gene expression was analyzed using the 2^-ΔΔct^ method. The expression of the 18S ribosomal RNA gene (unigene comp170_c0, 100% similarity) was stable in the four developmental stages based on the RNA-seq data; this gene was therefore used as the internal reference (S2 Table).

## Results

### Four developmental stages of *H*. *marmoreus*

**Fig 1:**
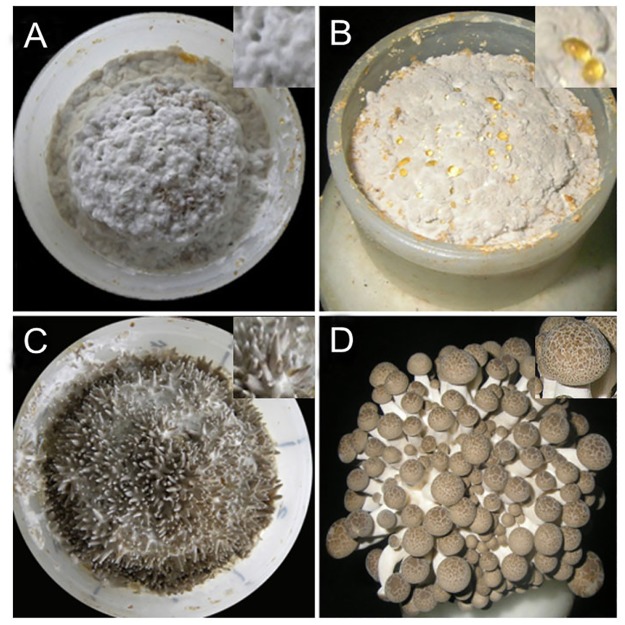
The different developmental stages of *H*. *marmoreus*. A: mycelial knot (H-M), B: mycelial pigmentation (H-V), C: primordium (H-P) and D: fruit body (H-F).

The developmental process of *H*. *marmoreus* can be divided into four main stages: the mycelial knot (H-M), mycelial pigmentation (H-V), primordium (H-V) and fruiting body (H-F) stages (Fig 1). The morphological changes under standard cultivation conditions were observed after mycelial maturation following the decrease in temperature and increase in light intensity (50–200 lux). Under these conditions, the mycelium turned into fluffy hyphal knots that were 0.5–1.0 mm in diameter and white in color (Fig 1A). The mycelium transformed from vegetative growth to reproductive growth, and the color changed to gray brown in 3–4 days (Fig 1B). After the mycelial pigmentation stage, the pinning fruit body appeared in 3–5 days at the primordium stage, as determined by morphology (Fig 1C). When the light intensity was increased to 200–1000 lux after 6–8 days, the fruit body matured, and the fruit bodies were composed of long stipes and closed caps, which were spotted with water-spots and had a creamy brown color [35–36]; these characteristics can be seen in Fig 1D.

### Illumina sequencing and de novo assembly

**Fig 2:**
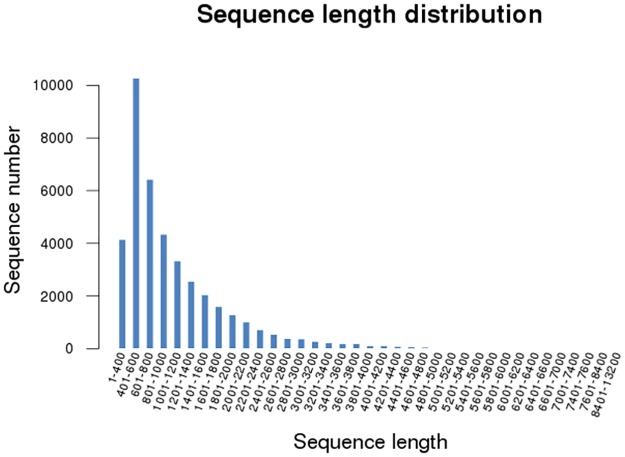
The length distribution of assembled *H*. *marmoreus* unigenes.

Each sample from the four *H*. *marmoreus* developmental stages produced over 1 Gbp of raw data (raw Gigabases) from paired-end (PE) reads, with a single read length of approximately 100 bp. After sequencing, over 59.3 million raw reads and 5.99 billion raw bases were obtained in a single sequencing run (Table 1), indicating that approximately 5.99 Gbp of sequenced data was obtained. The sequencing depth was tested [37], and the results (S1 Fig) indicated that the sequencing saturation of the four samples was high and that the sequencing capacity covered most of the expressed *H*. *marmoreus* genes. Unigenes from each sample were assembled using the sequence clustering software Trinity, resulting in 40568 unigenes with a mean length of 1074 bp and the lengths of the majority unigenes were in the 401–600 bp range (Table 2 and Fig 2). To complete the sequencing of the *H*. *marmoreus* transcriptome, the reads were mapped to the assembled unigenes, and the mapping results are shown in S3 Table.

**Table 1:** The reads and bases numbers for *H*. *marmoreus* transcriptome.

Samples	Reads (raw data/after cleaning)	Base No (raw data/after cleaning)
**H-M**	19780340/18847418	1997814340/ 1758550929
**H-V**	10535806/10044633	1064116406/ 928316621
**H-P**	17221464/16391924	1739367864/ 1524438387
**H-F**	11719956/11215472	1183715556/ 1032250851
**Total**	59257566/56499445	5985014166/5243556788

**Table 2:** The assembled results for *H*. *marmoreus* transcriptome.

Type	Number
**Total gene**	14321
**Total unigenes**	40568
**Total residues**	43609521
**Average length**	1074.97
**Largest unigene**	13082
**Smallest unigene**	351
**Unigenes for H-M**	26949
**Unigenes for H-V**	27541
**Unigenes for H-P**	27895
**Unigenes for H-F**	26506

### Functional annotation of *H*. *marmoreus* transcriptome

Of the unigenes, 29757 (73.35%) were predicted ORFs. The amino acid sequences of the predicted ORF were annotated using BLASTp against NR (non-redundant protein sequences), string and gene databases, with a cut-off E-value of 10^-5^. The nucleotide sequences of the unpredicted ORFs were annotated using BLASTx against NR, string and gene databases, with the same cut-off E-value. Of the 29757 predicted and 10811 unpredicted ORFs, 26800 (66.06%) and 2895 (26.78%) were blasted against public databases, respectively, and 7.16% ORFs were found not to match proteins from public databases. A list of the results from blasting *H*. *marmoreus* unigenes against de novo transcriptomes and ESTs of other fungi from public databases are shown in S1 Dataset, and the proportional distribution of sequences matching to the top ten basidiomycetes are shown in S2 Fig. The *H*. *marmoreus* transcriptome produced a strong match against the *L*. *bicolor* genome.

**Fig 3:**
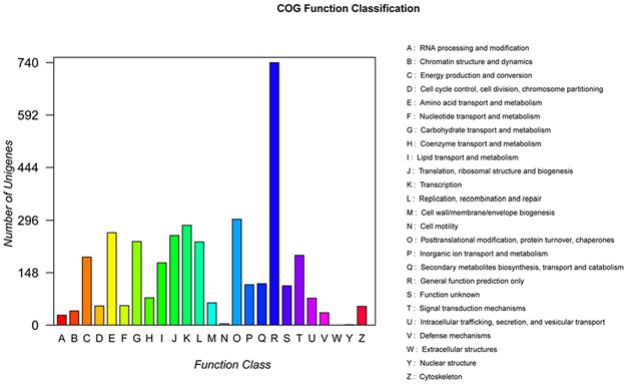
COG functional categories of *H*. *marmoreus* unigenes.

To further evaluate the effectiveness of the annotation process and the completeness of the transcriptome, we searched the annotated sequences for unigenes involved in Clusters of Orthologous Groups of proteins (COGs) classifications. A total of 4641 unigenes were classified into at least 25 functional groups (Fig 3). The cluster for “General function prediction only” represented the largest group, followed by “Posttranslational modification, protein turnover, chaperones” and “Transcription”. Meanwhile, a total of 3618 unigenes were assigned to 25 KOG functional categories (S3 Fig).

**Fig 4:**
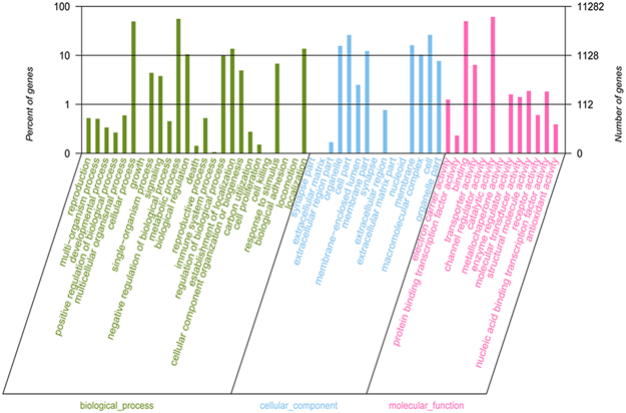
Gene Ontology classification of the *H*. *marmoreus* transcriptome. Histogram of the GO annotation was generated by the Blast2GO software (http://www.blast2go.com/b2ghome). The unigenes were grouped into three main GO categories: cellular component, molecular function and biological process. The right Y-axis indicates the number of unigenes in a category. The left Y-axis indicates the percentage of a specific category. One unigene could be assigned with more than one GO term.

We used Gene Ontology (GO) assignments to classify the functions of the annotated unigenes to other fungi, and these unigenes also strongly matched the *L*. *bicolor* genome. Of the annotated unigenes, 11282 were categorized into 53 functional groups (Fig 4), with 25 involved in biological process, 15 involved in cellular component and 13 involved in molecular function. Most of the corresponding genes were involved in “Cellular and metabolic processes” in the categories of biological process. In the cellular component categories, most of the corresponding genes were involved in “Cell”, “Cell part” and “Membrane”. In the molecular function categories, most of the corresponding genes were involved in “Binding” and “Catalytic activity”. Forty-five unigenes were assigned to the ‘Developmental process’ and ‘Growth’ categories, and the expression patterns of these unigenes are shown in S4 Fig. In addition, 15565 unigenes are annotated in the Kyoto Encyclopedia of Genes and Genomes (KEGG) and mapped to 305 reference canonical KEGG pathways.

Analysis of differential expression genes (DEGs) in the four developmental stages

**Fig 5:**
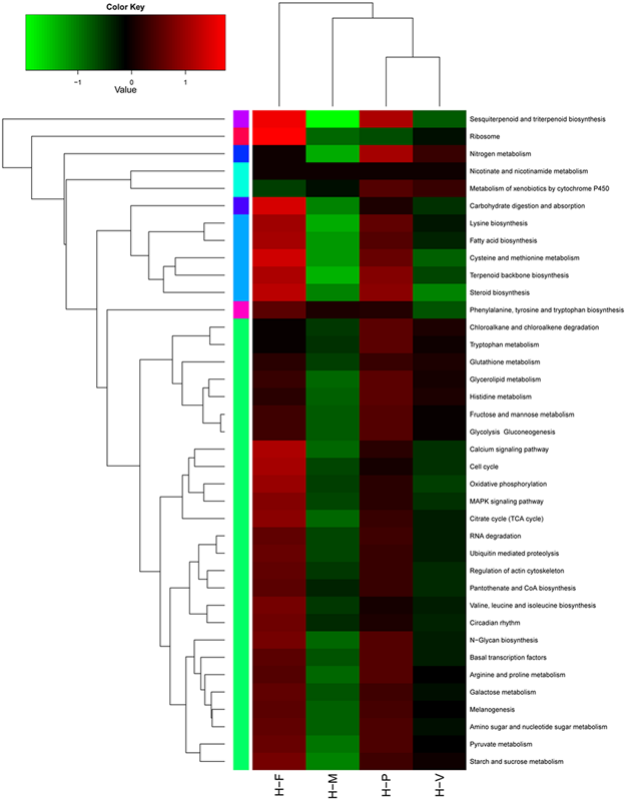
KEGG annotation of DGEs. The heat map shows 38 annotated pathways of DGEs in the mycelial knot (H-M), mycelial pigmentation (H-V), primordium (H-P) and fruiting body (H-F) stages of *H*. *marmoreus*. Different colors represent different expression levels. Green represents down-regulated expression and red represents up-regulated expression. Each row represents a different pathway. The heat map was constructed based on the log10 values of the RPKM of all unigenes related to a particular pathway in the four developmental stages.

To study the function of differential expression genes (DEGs), functional annotation was adopted for these identified genes. The metabolic and regulatory pathways related to the DEGs were analyzed and the heatmap analysis was performed based on the total FPKM values of all the DEGs in each pathway (Fig 5). Among these pathways, most were up-regulated in the H-P and H-F stages. These pathways included “Ribosome”, “Nitrogen metabolism”, “Ca^2+^ signaling pathway”, “MAPK signaling pathway”, “N−Glycan biosynthesis”, “Ubiquitin mediated proteolysis” and “Amino sugar and nucleotide sugar metabolism”. Only the “Phenylalanine, Tyrosine and tryptophan biosynthesis” pathway was active in H-M, and the “Melanogenesis”, “Glycerolipid metabolism”, “Glutathione metabolism” and “Histidine metabolism” pathways were up-regulated in H-V.

**Fig 6:**
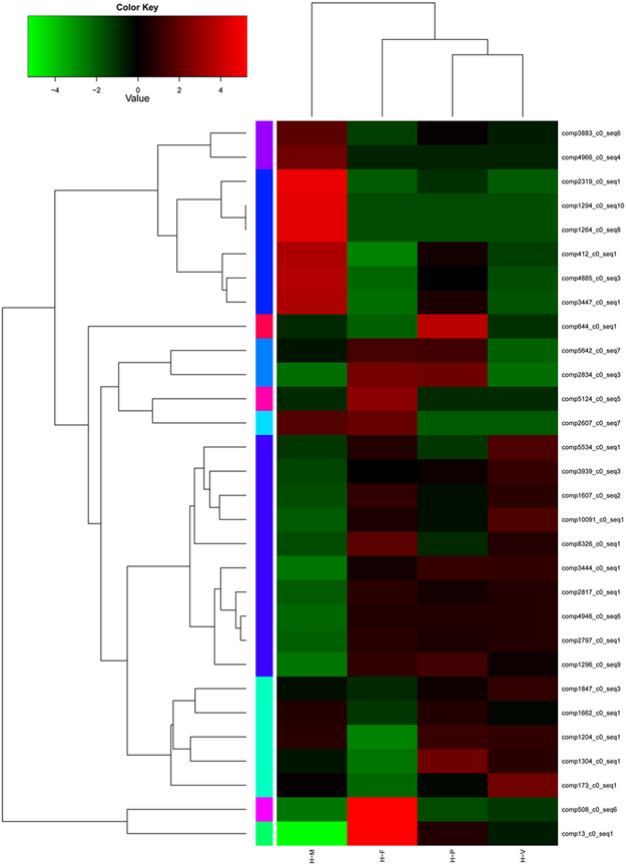
Differentially expressed genes (DEGs) between the four developmental stages in *H*. *marmoreus*. Each column represents an experimental sample (e.g., H-M, H-V, H-P and H-F), and each row represent a gene. Expression differences are shown in different colors. Red indicates high expression and green indicates low expression.

Co-expression analysis, which is based on the premise that a set of genes involved in a biological process is co-expressed under given conditions, has been successfully used to identify novel genes in several studies [38]. To verify the correlation between the expression of differentially expressed genes (DEGs) and developmental stages, heatmap analysis was performed based on the FPKM values of 31 DEGs involved in metabolism, gene regulation, signal transduction and cell organization (Fig 6). The functional annotation for these unigenes are listed in S4 Table. As shown in Fig 6, genes involved in carbon metabolism, such as the unigenes manganese peroxidase (comp412_c0_seq1) and laccase (comp3447_c0_seq1), had higher expression in H-M. The gene encoding tyrosinase (comp1847_c0_seq3) exhibited the highest expression level in H-V. In H-P, different unigenes, such as Precursor priA (comp1204_c0_seq1), adenylate kinase (comp1662_c0_seq1), RhoA GTPase effector (comp2834_c0_seq3) and serine threonine protein kinase (comp3444_c0_seq1) were up-regulated. In H-F, the genes encoding Ca-transporting ATPase (comp2607_c0_seq7), Ras GTPase-activating protein (comp2797_c0_seq1) and AMP binding protein (comp5124_c0_seq5) were up-regulated.

**Fig 7:**
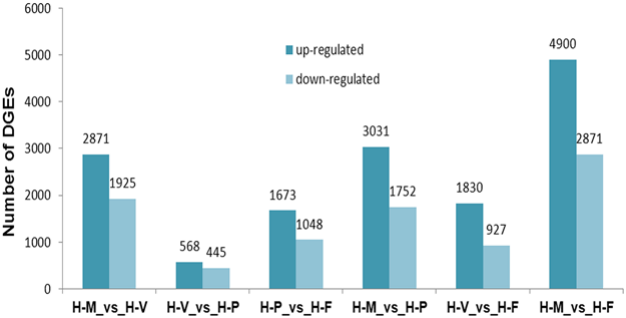
Numbers of differentially expressed unigenes in each comparison.

The total RNA from the H-M, H-V, H-P and H-F stages were used to construct different pairwise comparisons of gene expression in the samples; six DEGs libraries were obtained (H-M to H-V, H-V to H-P, H-P to H-F, H-V to H-F, H-M to H-P and H-M to H-F). We analyzed the DEGs between H-M to H-V and identified a total of 4796 unigenes, including 2871 genes that were up-regulated in the H-M stage and 1925 genes that were up-regulated in H-V (Fig 7 and S2 Dataset). There were 1526 unigenes that were expressed only in H-M, and 821 unigenes were expressed specifically in H-V. Based on the GO functional enrichment analysis of all the DEGs between H-M to H-V, the main GO terms were enriched in “Carbohydrate metabolic process”, “Lignin metabolic process”, “Cellulose metabolic process”, “Polysaccharide metabolic process”, “Peroxidase reaction” and “Oxidoreductase activity”. Based on the KEGG enrichment analysis of all the DEGs between H-M to H-V, 225 pathways were enriched, and 6 gene sets were significantly enriched. Most of these genes were up-regulated in the H-V library and correlated to “Starch and sucrose metabolism”, “Lysosome”, “Methane metabolism” and “Amino sugar and nucleotide sugar metabolism”.

A total of 1013 DEGs, including 568 up-regulated in H-V and 445 up-regulated in H-P were identified in the transition from H-V to H-P (Fig 7 and S3 Dataset). Among the 1013 DEGs, 215 DEGs showed specific expression in H-V, and 259 DEGs showed specific expression in H-P. Based on the GO functional enrichment analysis of all the DEGs between H-V to H-P, unigenes related to “Cellobiose glucosidase activity”, “beta-glucosidase activity”, “Cellular carbohydrate metabolic process”, “UDP-N-acetylmuramate dehydrogenase activity”, “Alcohol metabolic process” and “Glucan catabolic process” were significantly enriched in the transition from H-V to H-P. Based on the KEGG enrichment analysis of all the DEGs between H-V to H-P, 122 pathways were enriched in the transition from H-V to H-P, and 7 genes sets were significantly enriched. Most of these genes were up-regulated in the H-P library and correlated to “Phenylpropanoid biosynthesis”, “Peroxisome”, “Oxidative phosphorylation” and “Pentose and glucuronate interconversions”.

There were 2721 DEGs identified between the H-P to H-F stages, including 1673 up-regulated genes in H-P and 1048 up-regulated genes in H-F (Fig 7 and S4 Dataset). Four hundred four unigenes showed only expression in H-P, and 251 unigenes showed specific expression in H-F. Based on the GO functional enrichment analysis of all the DEGs between H-P to H-F, unigenes related to “Translation”, “Protein-DNA complex”, “Transport”, “Ribosome biogenesis” and “Nucleosome” were significantly expressed during the transition from primordium to fruit body. Based on the KEGG enrichment analysis of all the DEGs between H-P to H-F, 188 pathways were enriched in the H-P to H-F database, and 16 gene sets were significantly enriched. Most of these genes were up-regulated in the H-F library and correlated to “Ribosome”, “mTOR signaling pathway”, “Histidine metabolism”, “Metabolism of xenobiotics by cytochrome P450” and “Fatty acid metabolism”.

### Pathways involved in the four developmental stages

**Fig 8:**
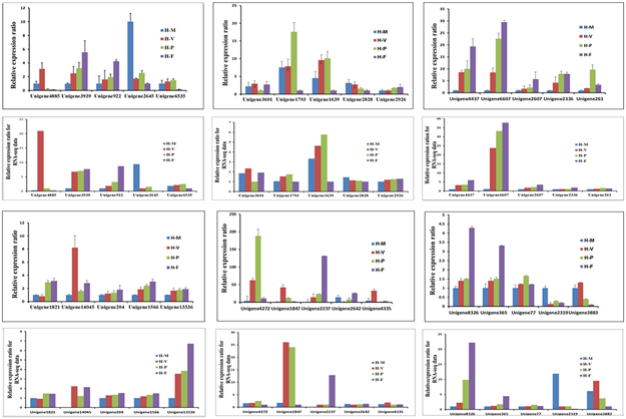
The qRT-PCR analysis of gene expression compared to the RNA-seq data in H-M (blue bars), H-V (red bars), H-P (green bars) and (purple bars). The Y-axis represents the relative expression levels of the four samples. The unit for the RNA-seq data is FPKM. The error bars represent the technical replicates of three independent replicates.

**Fig 9:**
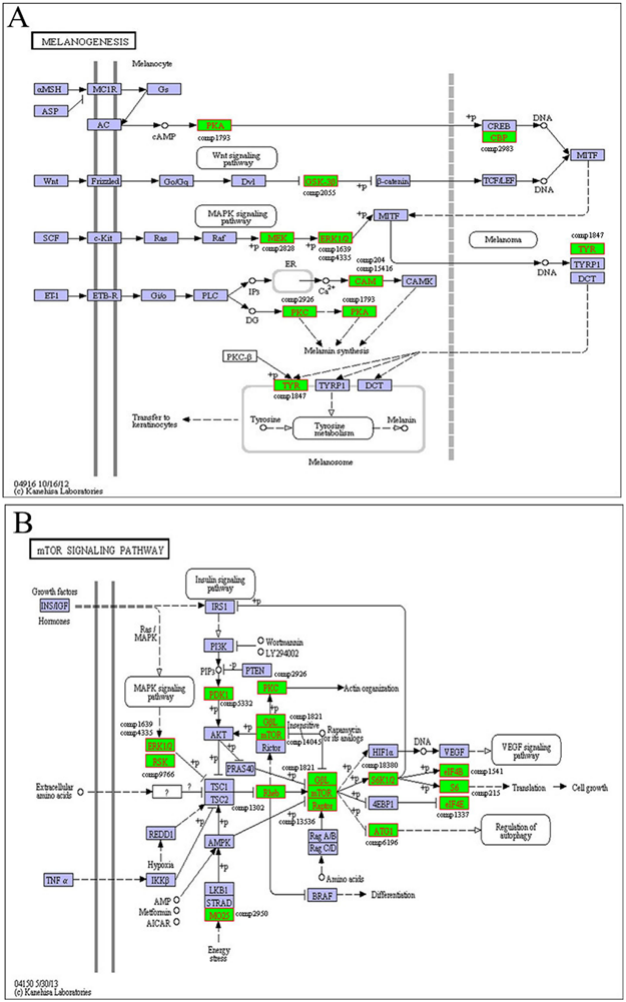
Mycelium pigmentation stage-related metabolic pathways (Fig 9A) (http://www.kegg.jp/kegg-bin/show_pathway?ko04916) and the mTOR signaling pathway (Fig 9B) (http://www.kegg.jp/kegg-bin/show_pathway?ko04150), identified by KEGG annotation. The green boxes indicate that the genes identified in the transcriptome of *H*. *marmoreus* are annotated in the metabolic pathways and the genes names were added in the figure.

In the *H*. *marmoreus* transcriptome, 10 unigenes, which were involved in the melanogenesis pathway [39], were identified in the stage of mycelium pigmentation (Table 3). The qRT-PCR results (Fig 8) revealed that the expression of the tyrosinase gene (comp1847_c0) was 41.8-fold higher in H-V than in H-M. The genes encoding MAPK, PKA and CAM, which are involved in the MAPK, cAMP and Ca^2+^ signaling pathways were also found in the melanogenesis pathway [39] (Fig 9A), which was determined using KEGG analysis. In addition, up-regulated genes in the transition from H-M to H-V included those encoding blue light receptor (blr), phytochrome-like protein, phytochrome-related signal transduction histidine kinase (comp922_c0, comp6535_c0 and comp4272_c0) (Fig 8), and three unigenes encoding histidine kinases (comp2881_c0, comp4547_c0 and comp4846_c0) that are involved in the two-component system. The KEGG enrichment analysis also revealed that the two-component system was enriched in the transition from H-M to H-V.

**Table 3:** *H*. *marmoreus* unigenes putatively associated with the mycelium pigmentation stage.

EST ID	Gene name	E-value	Accession no.	Organism	KEGG ID
**comp1847**	tyrosinase	6e-36	XP_001885552	L. bicolor	K00505
**comp204**	calmodulin	0	BAM24398	P. chrysosporium	K02183
**comp15416**	calmodulin	0	XP_002998616	P. ainfestans	K02183
**comp2828**	mitogen-activated kinase protein	0	EIW55669	T. versicolor	K04368
**comp4335**	mitogen-activated kinase protein	0	EJF57847	D. squalens	K04371
**comp1639**	mitogen activated kinase protein	0	EGO03785	S. lacrymans	K04371
**comp2983**	CBP	2e-16	XP_001884277	L. bicolor	K12882
**comp1793**	protein kinase A	2e-147	XM_001829981	C. cinerea	K04345
**comp2926**	protein kinase C	0	XP_001876242	L. bicolor	K02677
**comp2055**	Gsk-3p	7e-145	YP_117550	N. farcinica	K03083

It was found that genes involved in the MAPK and cAMP signaling pathways, as well NADPH oxidase enzymes, were up-regulated in the transition from H-V to H-P. As shown in Fig 8, genes encoding PKA (comp1793_c0), Mkk1_2 (comp1556_c0), Pbs2 (comp4437_c0) and Noxs (comp2336_c0 and comp261_c0) were all up-regulated in the transition from H-M to H-P. The gene encoding PKC (comp2926_c0) was up-regulated in the transition from H-V to H-P. NADPH oxidase genes *NoxB* (comp2336_c0) and *NoxR* (comp261_c0) were also up-regulated in H-P compared to H-V (Fig 8).

Nitrogen metabolism, mTOR signaling, Ca^2+^ signaling and pheromone reactions were up-regulated during the transition from H-P to H-F. Genes encoding ammonium transporter (ats, comp2237_c0), nitrate reductase (nrd, comp6607_c0), mTOR (comp14045_c0), Ca^2+^-ATPase (comp2607_c0) and STE3-like pheromone receptor (comp8326_c0), which are involved in mTOR signaling (Fig 9B and S5 Table), Ca^2+^ signaling (S5 Fig) and the MAPK pathway (S6 Fig), respectively, were up-regulated from H-P to H-F (Fig 8). Higher light intensity (200–1000 lux) was needed to induce fruit body maturation than was required before primordium initiation (50–100 lux) in *H*. *marmoreus*. It was found that the blue light receptor (comp922_c0), one of the central components in the blue-light signal transduction pathway, was up-regulated 2.7-fold in H-F than in H-P based on qRT-PCR results (Fig 8).

### Validation of transcriptome data by qRT-PCR

The expression profiles obtained by RNA-seq were validated by qRT-PCR analyses for 30 genes of interest (Fig 8), including those associated with the mycelium pigmentation, the MAPK pathway, cAMP signaling, the P450 gene family (S7 Fig) mTOR signaling, etc. The expression levels of these genes in the RNA-seq data are also shown in Fig 8. In all cases, the qRT-PCR data were consistent with sequencing results (Fig 8).

## Discussion

*H*. *marmoreus* is a very important mushroom species, primarily due to its organoleptic [8] and medicinal [9] properties. Recent research has shown that *H*. *marmoreus* is a useful organism for studying the developmental process of mushroom morphogenesis [40–41]. However, little is known regarding the mechanism of H-F development in this species, and genomic information for *H*. *marmoreus* is unavailable. The aims of this study were (i) to generate a large amount of cDNA sequence data that could facilitate more detailed studies in *H*. *marmoreus* and (ii) to identify the genes associated with the H-F process. The transcriptome data of *H*. *marmoreus* will be valuable for functional studies of this species and its relatives.

### Genes related to light response in the developmental stages

Light-induced brown mycelium formation is an important step during development in some mushrooms and is associated with the hyphal aggregation that precedes the formation of primordia [42–43]. In *Lentinula edodes*, the *Le*. *phrA* gene was most abundantly transcribed in the immature fruiting body, and the expression of *Le*. *phrB* was induced by light exposure in pre-primordial mycelia [44–45]. Tang et al. [46] found the expression levels of WC-1 and WC-2, together with phytochrome, were significantly up-regulated in brown mycelia in *L*. *edodes*. In *C*. *cinerea*, white collar proteins also play a role in photomorphogenesis and fruit body development [7–8]. In this study, genes encoding the blue light receptor (comp922_c0), phytochrome-like protein (comp6535_c0) and phytochrome-related signal transduction histidine kinase (comp4272_c0) were up-regulated in the transition from H-M to H-V (Fig 8). This result is consistent with previous studies in *L*. *edodes* [44–46] and *C*. *cinerea* [7–8]. At the early developmental stage, a light intensity of 50–200 lux was needed to induce fruit body initiation in *H*. *marmoreus*. These results indicated that blue light receptor and phytochrome might be associated with light-induced primordium initiation in *H*. *marmoreus*.

Genes encoding tyrosinase (comp1847_c0) was found to be up-regulated 41.8-fold in H-V compared with H-M (Fig 8). Tyrosinases are involved in the mycelium pigmentation stage and are responsible for the first step of melanin synthesis, namely, from L-tyrosine to the formation of L-dopaquinone and L-dopachrome [47]. In *A*. *bisporus*, tyrosinases play crucial roles in the formation of brown-colored melanin during developmental stages and after harvest [48]. In addition, the occurrence of fruiting bodies in mushrooms is facilitated by light. The use of blue light for oak mushroom has been shown to induce pigmentation in the primordial stage [49]. In *H*. *marmoreus*, the color of mycelia was gray-brown, and a great deal of pigment formed during the H-V stage (Fig 1B). The up-regulated expression of tyrosinase gene (comp1847_c0) and the formation of pigment in H-V suggested that tyrosinase was important in the transition from H-M to H-V of *H*. *marmoreus* under light stimulation.

### Genes related to stress response in the developmental stages

Fruit body development is often induced after the environment is drastically altered [1]. MAPKs that transmit environmental stress signals are also known as stress-activated protein kinases (SAPKs). In multicellular fungi, genes that are homologous to MAP kinases are involved in development and hyphal growth [50–51]. The qRT-PCR results revealed that the genes encoding Mkk1_2 (comp1566_c0), Pbs2 (comp4437_c0), which are important in the MAPK cascades that are involved in starvation and hypotonic shock reactions, had higher expression levels in H-P than in H-M (Fig 8). These observations are consistent with previous findings in *C*. *cinerea* for which it was reported that two MAPK cascades were up-regulated in the primordium stage relative to the mycelium stage [22]. These results together suggest that the MAPK signaling pathway might be associated with primordium initiation in *H*. *marmoreus*.

Multiple Nox isoforms are present in multicellular eukaryotes [52]. Fungal Nox enzymes were first discovered in filamentous fungi [53]. Recent research has indicated that Nox enzymes play important roles in regulating the development of fungi. In *A*. *nidulans*, *Podospora anserine* and *N*. *crassa*, NoxA (Nox1) and NoxB (Nox2) are required for ascospore development, indicating that a major function of the two isoforms is to regulate the development of multicellular organisms [54–56]. Mu et al. [6] found that Nox enzymes regulate hyphal branching and fruiting body development in *Ganoderma lucidum*. In the present study, NoxB (comp2336_c0) and NoxR (comp261_c0) were up-regulated in H-P relative to H-V, suggesting that the NADPH oxidase gene family might also be associated with the initiation of the primordium stage in *H*. *marmoreus*.

### Genes related to nitrogen starvation in the developmental stages

**Fig 10:**
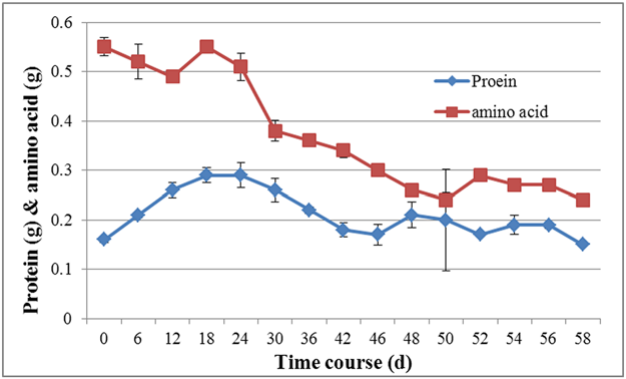
Changes of soluble protein and free amino acids in liquid medium of *H*. *marmoreus*. 0–36: mycelium stage; 36–48: primordium stage; 48–58: fruiting body stage. The data represent average values of triplicate cultures with standard error of the mean.

Fruiting body formation is very much influenced by the physiological condition and nutritional state of the mycelium [57]. Nitrogen shortage in the mycorrhizosphere is believed to favor the establishment of the ectomycorrhizal symbiosis [58–60]. In basidiomycetes, low levels of nitrogen may be commercially beneficial because this condition satisfies the needs of fungi for growth and fruiting but can inhibit microbial competitors. The genes encoding ammonium transporter (comp2237_c0) and nitrate reductase (comp6607_c0) were up-regulated in H-F, as was the ribosomal protein (comp365_c0). As shown in Fig 10, the protein (BACTM protein kit) [61] and free amino acid concentrations (A200 amino acid analyser) [62] in the liquid medium reached their lowest levels in H-F] in *H*. *marmoreus*. Our results agreed with previous work finding that nitrogen metabolism is an important factor in regulating morphogenesis in *A*. *bisporus* [24]. Furthermore, mTOR (comp14045_c0) and its associated protein Raptor (comp13536_c0), which can respond to changes in nutrient conditions, were up-expressed from H-P to H-F. In *C*. *cinerea*, Cheng et al. [22] found that mTOR signaling pathway functions are essential regulators during fruiting body formation. These results suggested that nitrogen starvation in *H*. *marmoreus* might be more important for fruit body maturation than in the other developmental stages.

## Conclusion

**Fig 11:**
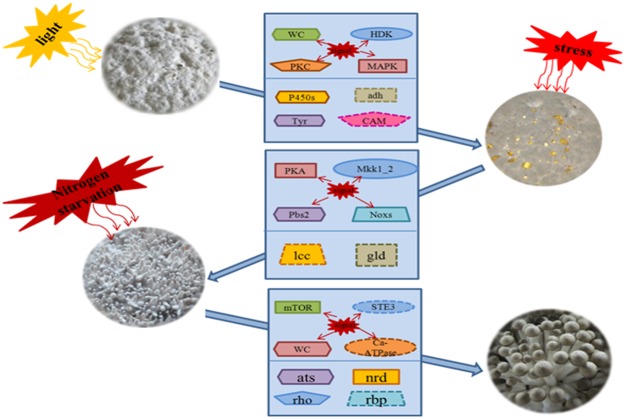
Pathways and genes associated with the three transitions between the four developmental stages in *H*. *marmoreus*. Light may play a crucial role during the first transition as determined by the potential involvement of tyrosinase (Tyr), the blue light transduction signal pathway (WC), the MAPK signaling pathway (MAPK), the cAMP signaling pathway (PKC) and the two-component system pathway (HDK). In addition, the cytochrome P450 (P450s) and alcohol dehydrogenase (adh) genes were active in this process to facilitate mycelial growth. During the second transition, pathways related to stress reactions, such as the MAPK signaling (Mkk1_2 and PBS2) and cAMP signaling (PKA) pathways, were active, as were NADPH oxidase enzyme (Noxs) and the genes encoding glucosidase (gld) and laccase (lcc). These pathways and genes might be associated with primordium initiation. During the last transition, pathways related to nitrogen starvation, such as nitrogen metabolism (ats, nrd), mTOR signaling (mTOR) and Ca^2+^ signaling (Ca^2+^-ATPase), might be more important in fruit body maturation than in other stages. Genes encoding ribosomal protein (rbp), rho guanine nucleotide exchange factor scd1 (rho) and STE3-like pheromone receptor (ste3) had higher expression levels in fruit body maturation than in the other stages. The solid line indicates that the results were confirmed by transcriptomic analysis and RT-PCR; the dotted line indicates that the results were only confirmed by transcriptomic analysis.

While *H*. *marmoreus* is an important edible mushroom species in Asia, the molecular/genetic basis of mushroom morphogenesis remains largely uncharacterized. In this work, the transcriptomes of four developmental stages of *H*. *marmoreus* were profiled and summarized in a schematic model (Fig 11). It was found that light affects initiation of the fruit body and that the expression of the genes encoding Tyr, MAPK, PKC, and BLR were altered during the mushroom initiation. In the transition from H-V to H-P, environmental stresses might be associated with primordium initiation, which leads to alterations in the expression of genes such as PKA, Pbs2 and Noxs. In addition, the carbon metabolism genes encoding glucose-6-P dehydrogenase, alcohol dehydrogenase, laccase and glucosidase were expressed at higher levels in the H-V and H-P stages than in the other stages. In addition, genes involved in nitrogen metabolism and mTOR signaling pathway were the most active during the transition from H-P to H-F. To the best of our knowledge, this is the first report that studied the transcriptome of *H*. *marmoreus*. These data may provide a valuable resource for further studies of this mushroom. Further functional characterization of the unigenes that were found to be associated with the regulation of the four development stages may lead to an in-depth understanding of the network that that regulates fruit body development.

## Supporting Information

S1 Dataset All unigenes from the *H*. *marmoreus* transcrptome. (XLSX)

S2 Dataset Differentially expressed genes (DEGs) between *H*. *marmoreus* mycelium and pigmentation mycelium. From the *H*. *marmoreus* mycelium and pigmentation mycelium, 4,796 unigenes were DEGs, of which 2,871 up-regulated in mycelum and 1,925 up-regulated in pigmentation and 1,526 unigenes showed specific expression patterns in mycelium and 821unigenes were specific in the pigmentation mycelium.
(XLSX)

S3 Dataset Differentially expressed genes (DEGs) between *H*. *marmoreus* pigmentation mycelium and primordium. From the *H*. *marmoreus* pigmentation mycelium and primordium, 1,013 unigenes were DEGs, of which 568 up-regulated in pigmentation mycelium and 445 up-regulated in primordium and 215 unigenes showed specific expression patterns in the pigmentation mycelium and 259 unigenes were specific in the primordium.
(XLSX)

S4 Dataset Differentially expressed genes (DEGs) between *H*. *marmoreus* primordium and fruiting body. From the *H*. *marmoreus* primordium and fruiting body, 2,721 unigenes were DEGs, of which 1,673 up-regulated in primordium and 1,048 up-regulated in fruiting body and 404 unigenes showed specific expression patterns in the primordium and 251 unigenes were specific in the fruiting body.
(XLSX)

S1 Fig Saturation analysis of genes expression levels for the four developmental stages in *H*. *marmoreus*. The abscissa represents a valid comparison of the percentage of reads and the vertical axis represents the deviation ratio with 15% between the expression levels on the sampling condition to final value. A: mycelial knot (H-M), B: mycelial pigmentation (H-V), C: primordium (H-P) and D: fruit body (H-F).
(TIF)

S2 Fig Species distribution among hits to sequences of the *H*. *marmoreus* transcriptome. The graph shows ten species that the *H*. *marmoreus* transcriptome sequences were most similar to.
(TIF)

S3 Fig KOG functional categories of *H*. *marmoreus* unigenes. (TIF)

S4 Fig The expression levels of 45 genes among the four developmental stages in *H*. *marmoreus* DGE. Each column represents an experimental sample (eg. H-M, H-V, H-P and H-F) and each row represent a gene. Expression differences are shown in different colors. Red means high expression and green means low expression.
(TIF)

S5 Fig Calcium signaling pathway identified by KEGG annotation (http://www.kegg.jp/kegg-bin/show_pathway?ko04020). The green boxes indicate that the genes identified in the transcriptome of *H*. *marmoreus* are annotated in the metabolic pathways and the genes names were added in the figure.
(TIF)

S6 Fig MAPK signaling pathway identified by KEGG annotation (http://www.kegg.jp/kegg-bin/show_pathway?ko04011). The green boxes indicate that the genes identified in the transcriptome of *H*. *marmoreus* are annotated in the metabolic pathways and the genes names were added in the figure.
(TIF)

S7 Fig The expression levels of cytochrome P450 genes among the four developmental stages in *H*. *marmoreus* DGE. Each column represents an experimental sample (eg. H-M, H-V, H-P and H-F) and each row represent a gene. Expression differences are shown in different colors. Red means high expression and green means low expression.
(TIF)

S1 Table Primer sets used for quantitative real-time PCR. (PDF)

S2 Table The expression level of 18S ribosomal RNA gene in the RNA-seq data. (PDF)

S3 Table The ratio of mapping in each sample. (PDF)

S4 Table The functional annotation for 31 unigenes from six DGE libraries. (PDF)

S5 Table The unigenes of *H*. *marmoreus* putatively involved in the mTOR signaling pathway. (PDF)
