# Supplementary figures and images for: Transcriptome Analysis and Its Application in Identifying Genes Associated with Fruiting Body Development in Basidiomycete Hypsizygus marmoreus
Source: PLoS One. 2015 Apr 2;10(4):e0123025. doi: 10.1371/journal.pone.0123025 (PMC4383556; doi:10.1371/journal.pone.0123025)

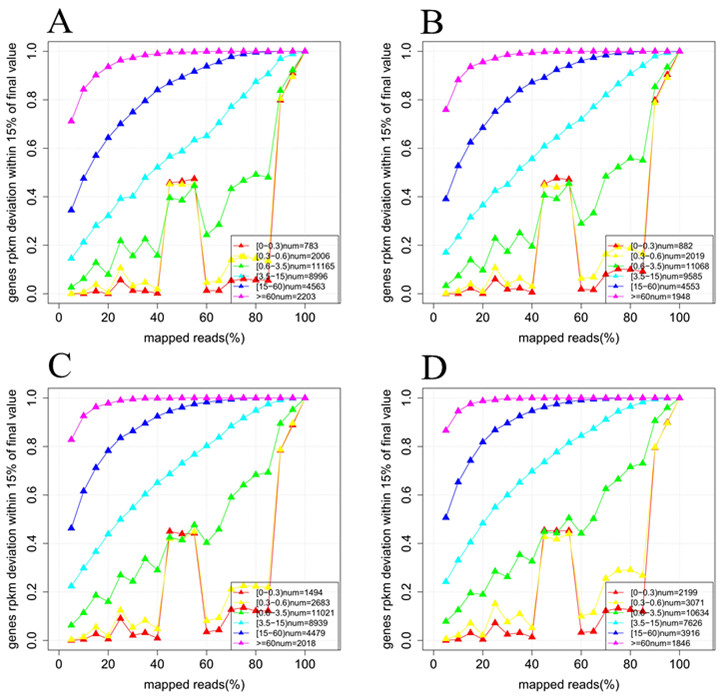

Supplement: S1 Fig — The abscissa represents a valid comparison of the percentage of reads and the vertical axis represents the deviation ratio with 15% between the expression levels on the sampling condition to final value. A: mycelial knot (H-M), B: mycelial pigmentation (H-V), C: primordium (H-P) and D: fruit body (H-F). (TIF) [file pone.0123025.s005.tif]

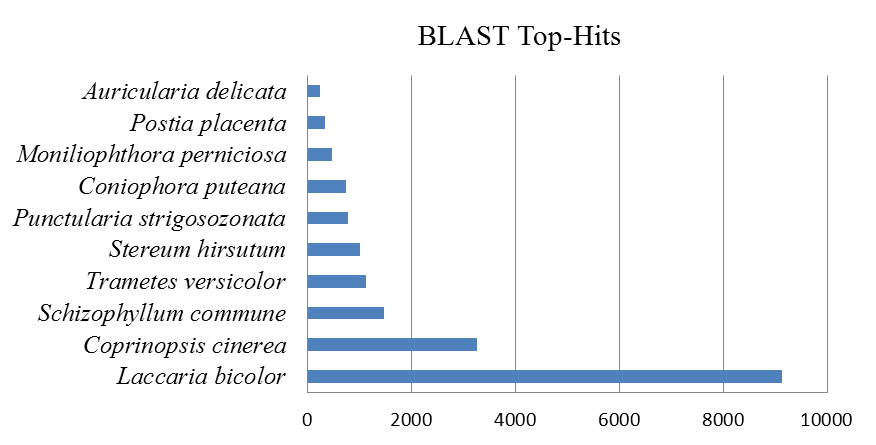

Supplement: S2 Fig — The graph shows ten species that the H. marmoreus transcriptome sequences were most similar to. (TIF) [file pone.0123025.s006.tif]

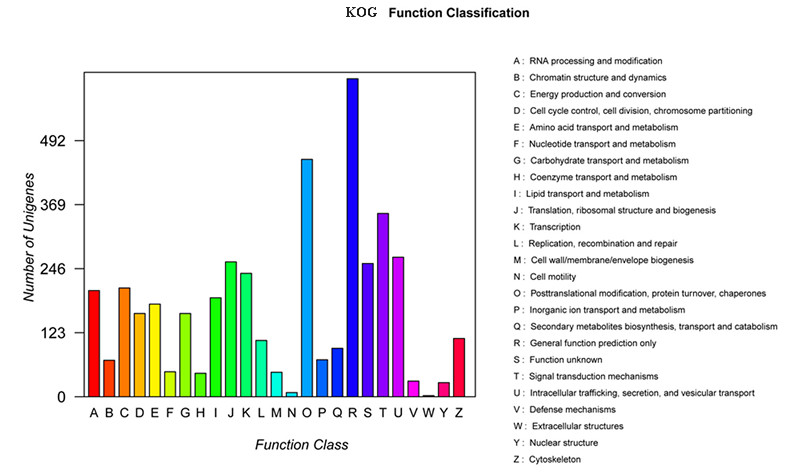

Supplement: S3 Fig — (TIF) [file pone.0123025.s007.tif]

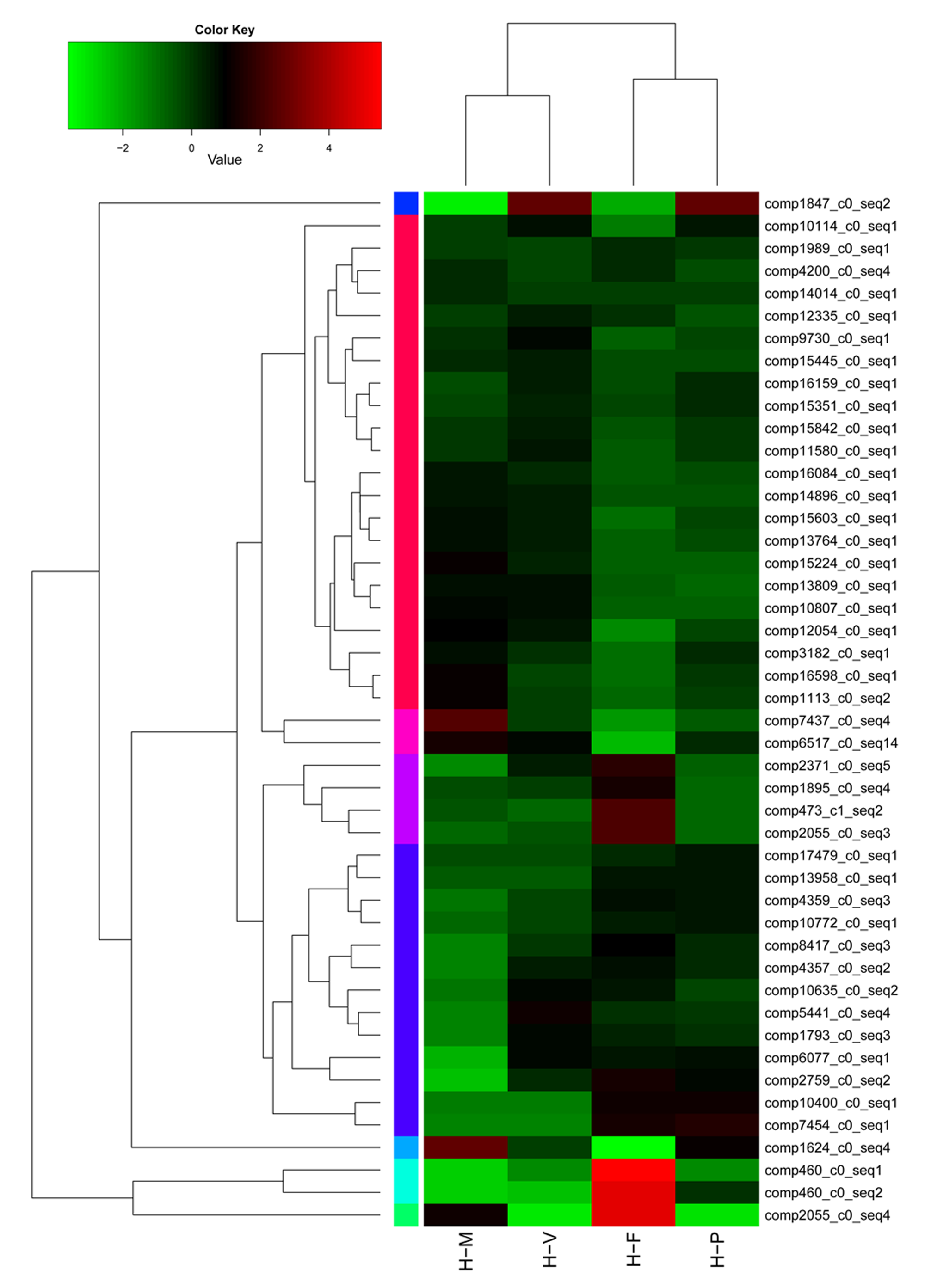

Supplement: S4 Fig — Each column represents an experimental sample (eg. H-M, H-V, H-P and H-F) and each row represent a gene. Expression differences are shown in different colors. Red means high expression and green means low expression. (TIF) [file pone.0123025.s008.tif]

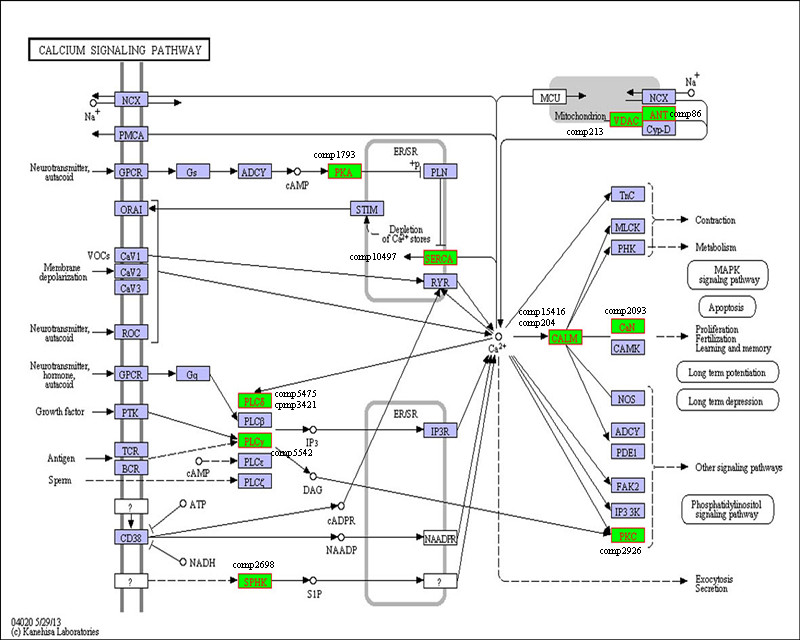

Supplement: S5 Fig — The green boxes indicate that the genes identified in the transcriptome of H. marmoreus are annotated in the metabolic pathways and the genes names were added in the figure. (TIF) [file pone.0123025.s009.tif]

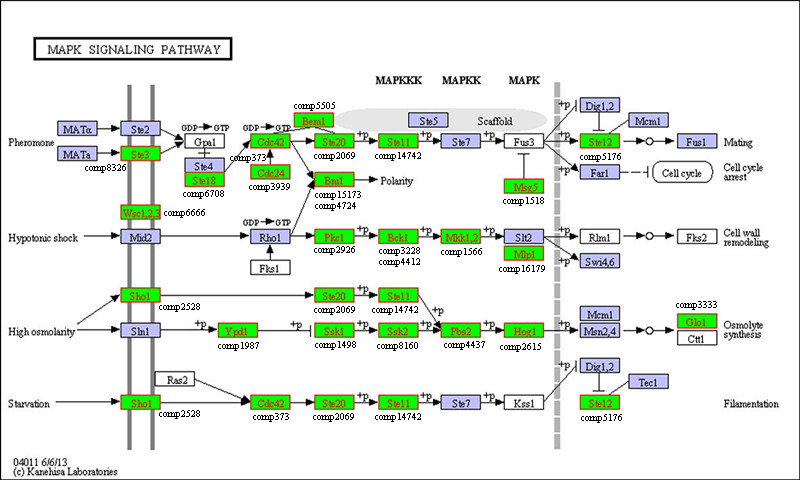

Supplement: S6 Fig — The green boxes indicate that the genes identified in the transcriptome of H. marmoreus are annotated in the metabolic pathways and the genes names were added in the figure. (TIF) [file pone.0123025.s010.tif]

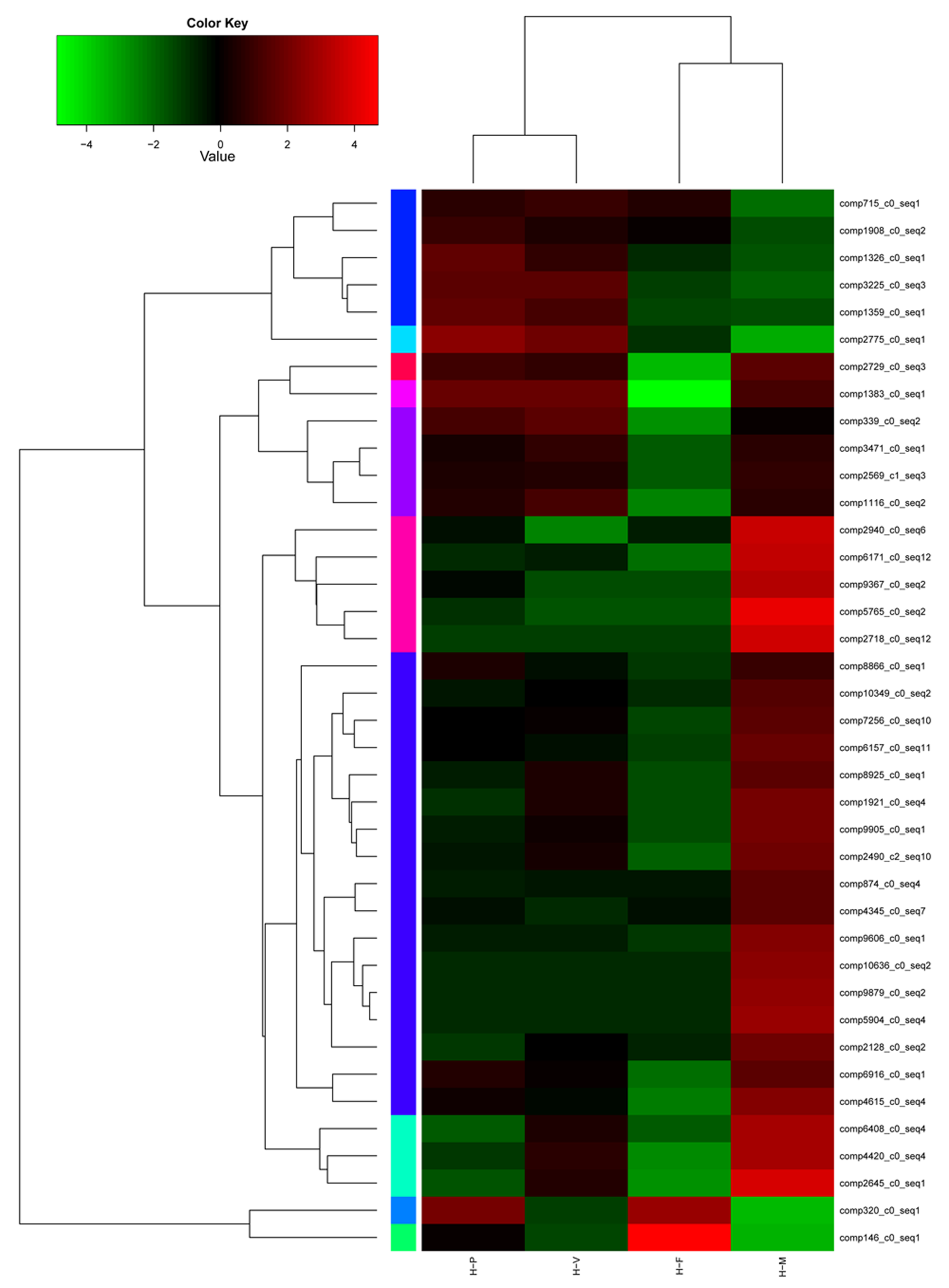

Supplement: S7 Fig — Each column represents an experimental sample (eg. H-M, H-V, H-P and H-F) and each row represent a gene. Expression differences are shown in different colors. Red means high expression and green means low expression. (TIF) [file pone.0123025.s011.tif]
