# Supplementary material for: Transcriptome Analysis and Its Application in Identifying Genes Associated with Fruiting Body Development in Basidiomycete Hypsizygus marmoreus
Source: PLoS One. 2015 Apr 2;10(4):e0123025. doi: 10.1371/journal.pone.0123025 (PMC4383556; doi:10.1371/journal.pone.0123025)
Supplement: S1 Table — (PDF) [file pone.0123025.s012.pdf]

S1_Table. Primer sets used for quantitative real-time PCR.

Primer name	Sequence (5'-3')	Accession No.
Unigene3601-F	AGCACCGCAGGCTCCAGTTA	KF705537
Unigene3601-R	ACGAGTAGCACCCGTCCAAA	
Unigene1793-F	AGATAGCCTCGGCGGGATGA	KF705538
Unigene1793-R	CAACTTCGGCGGCGTAGAAT	
Unigene1639-F	CCCTCGATGAGTTCTATGC	KF705539
Unigene1639-R	AATGTCAGCGTCTTGGTTAG	
Unigene2828-F	GGGGCAAGGAAACGGTGGAA	KF705540
Unigene2828-R	ATTGGGATCGGCGAGGAAGG	
Unigene2926-F	GGATAATGGACAAGGAGGATGG	KF705541
Unigene2926-R	GCAGTTGGAGTTCCGAAAGTCAT	
Unigene4272-F	GATGAGGGCACCCGATTT	KF705542
Unigene4272-R	GTTGGCTGTCTTGCGTCT	
Unigene1847-F	CGTGAAGAATGTGGGTATAAAGG	KF705543
Unigene1847-R	GGTCTCCAGGACTAGAATAAGTGTC	
Unigene2237-F	ACGCCCGTCCACATCTCA	KF705544
Unigene2237-R	TCGCAGACAGAGCAGAGCC	
Unigene2642-F	CAGAGCGGAATAAAGGAA	KF705545
Unigene2642-R	GAGCCAGTGAATCAGCAT	
Unigene4335-F	TATCCTCCTCCATCCATAACCAC	KF705546
Unigene4335-R	CATACGACACCATAAGCACCCTC	
Unigene8326-F	GGACGAAGCCCGTAAACA	KF705547
Unigene8326-R	CGATGCGAAGGAGTCAGC	
Unigene365-F	GTCAAGAGCGAGGACCAGA	KF705548
Unigene365-R	ATAGTCGCAGCGGTGATG	
Unigene77-F	GATCACCTCCAACTACTGC	KF705549
Unigene77-R	GAGCGACATAACAACACTTC	
Unigene2319-F	AGGAACTTCTCGCCAAAT	KF705550
Unigene2319-R	TACCTAACGCCCTACTCG	
Unigene3883-F	GCTATTCCTGTCGCCGTTGT	KF705551
Unigene3883-R	ATGGCATTGGTGTTTGAGATTG	
Unigene4885- F	GCCGTCATCACCATTTCC	KF705552
Unigene4885-R	CGAGGGCGAGTATAGCAGT	
Unigene3939-F	GATTCGCACTGACAGGGTC	KF705553
Unigene3939-R	GATGGCGGTTACGGTGTT	
Unigene922-F	TTAGGGCACGCACGAAGG	KF705554
Unigene922-R	ACAACATCCCGCCACTCC	
Unigene2645-F	GACGCTTCGGTCTTCATT	KF705555
Unigene2645-R	CTTTCTGGGATCGTGGTT	
Unigene6535-F	GACCAAGCGACTCGTTTAT	KF705556
Unigene6535-R	TCTGGAGTGTATTTGAGGGA	
Unigene1821-F	CACGGGAAGTGAGGATGG	KF705557
Unigene1821-R	CCCTGGTTTGGGTGAATA	

Unigene14045-F	GCATACGCTGCATTGGTG	KF705558
Unigene14045-R	TTGTGGGAGGGCATTGTG	
Unigene204-F	GAGGCGTTCAAGGTGTTC	KF705559
Unigene204-R	CTTCCCGAATCATCTCGT	
Unigene1566-F	GTATCCATAATTTCCTCCACC	KF705560
Unigene1566-R	ATCGCTCCTCGCTCTTTT	
Unigene13536-F	GCCTTTGGTGGTCTTGTG	KF705561
Unigene13536-R	ATTTCAGCGATTTGTCCC	
Unigene4437-F	CGGCGTATCAGGACAACT	KF705562
Unigene4437-R	GGACGAGACCGTGTAAGTG	
Unigene6607-F	AATGGCGAGGTTTATGAC	KF705563
Unigene6607-R	TTTAGACTTCCGATGTGGA	
Unigene2607-F	ACCGTCAAGAAACACTACCTC	KF705564
Unigene2607-R	TCATCACAACCTGCACCC	
Unigene2336-F	TTTTCAGTATCCCTGTTGG	KF705565
Unigene2336-R	TTCTGTGAAGGCATCTCC	
Unigene261-F	TGTTCGGCGGTGTTATTT	KF705566
Unigene261-R	ACGTTGGAGGGATGCTGT	
18s-F	GAGGGACCTGAGAAACG	KC510993
18s-R	ATAAGACCCGAAAGAGCC	
