# Supplementary material for: Transcriptome Analysis and Its Application in Identifying Genes Associated with Fruiting Body Development in Basidiomycete Hypsizygus marmoreus
Source: PLoS One. 2015 Apr 2;10(4):e0123025. doi: 10.1371/journal.pone.0123025 (PMC4383556; doi:10.1371/journal.pone.0123025)
Supplement: S2 Table — (PDF) [file pone.0123025.s013.pdf]

S2_Table. The expression level of 18S ribosomal RNA gene in the RNA-seq data.

Sample	Count No	RPKM
**H-M**	12743	1299.25
**H-V**	16269	1189.23
**H-P**	16623	821.02
**H-F**	14345	713.11
