# Supplementary material for: Transcriptome Analysis and Its Application in Identifying Genes Associated with Fruiting Body Development in Basidiomycete Hypsizygus marmoreus
Source: PLoS One. 2015 Apr 2;10(4):e0123025. doi: 10.1371/journal.pone.0123025 (PMC4383556; doi:10.1371/journal.pone.0123025)
Supplement: S3 Table — (PDF) [file pone.0123025.s014.pdf]

S3_Table. The ratio of mapping in each sample.

Sample	Reads No（pair）	Percentage (%)
H-F	14137348/18020034	78.45%
H-M	6422523/9599462	66.90%
H-P	11930924/15629148	76.34%
H-V	8116009/10768726	75.37%
