# Supplementary material for: Transcriptome Analysis and Its Application in Identifying Genes Associated with Fruiting Body Development in Basidiomycete Hypsizygus marmoreus
Source: PLoS One. 2015 Apr 2;10(4):e0123025. doi: 10.1371/journal.pone.0123025 (PMC4383556; doi:10.1371/journal.pone.0123025)
Supplement: S4 Table — (PDF) [file pone.0123025.s015.pdf]

S4_Table. The functional annotation for 31 unigenes from six DGE libraries.

EST ID	Gene function	E-value	Accession no.	Organism
comp13_c0_seq1	hydrophobin	0	XP_001878874	L. bicolor
comp173_c0_seq1	hydrophobin	0	BAB84546	P. nameko
comp1304_c0_seq1	beta-1,3-glucanase	0	BAE20245	L. edode
comp2607_c0_seq7	Ca-transporting ATPase	0	EIN0643	P. strigosozonata
comp2834_c0_seq3	RhoA GTPase effector	0	XP_001877017	L. bicolor
comp3447_c0_seq1	laccase	0	ABY78033	H. marmoreus
comp412_c0_seq1	manganese peroxidase	0	BAA33449	P. ostreatus
comp4885_c0_seq3	hydrophobin	4.00E-25	EJF63706	D. squalens
comp1204_c0_seq1	Protein priA	0	Q01200	L. edodes
comp1264_c0_seq8	activator of Hsp70 and Hsp90 chaperones	0	XP_001877221	L.bicolor
comp1294_c0_seq10	glucose-6-P dehydrogenase	0	XP_001876685	L. bicolor
comp1296_c0_seq9	expressed protein	0	XP_003035340	S. commune
comp2319_c0_seq1	alcohol dehydrogenase	0	EIN11780	P. strigosozonata
comp2797_c0_seq1	Ras GTPase activating protein	0	XP_001836503	C. cinerea
comp2817_c0_seq1	chitin synthase	0	BAF41225	P. ostreatus
comp3444_c0_seq1	serine threonine protein kinase	0	EHK25855	T. virens
comp4946_c0_seq6	actin-related protein	0	XP_001874573	L. bicolor
comp4966_c0_seq4	CMGC/SRPK protein kinase	0	XP_001828589	C. cinerea
comp508_c0_seq6	hydrophobic surface binding protein	0	XP_001882714	L. bicolor
comp5124_c0_seq5	AMP binding protein	0	XP_001830320	C. cinerea
comp644_c0_seq1	terpenoid synthase	3.00E-38	EJF61156	D.squalens
comp8326_c0_seq1	STE3-like pheromone receptor	0	XP_001888610	L.bicolor
comp10091_c0_seq1	CAMK/CAMKL/PASK protein kinase	0	XP_001830169	C.cinerea
comp1607_c0_seq2	beta-glucan synthase	0	XP_001878782	L. bicolor
comp1662_c0_seq1	adenylate kinase	0	XP_001833219	C. cinerea
comp3883_c0_seq6	serine proteinase 2	0	CAC83024	A. bisporus
comp5534_c0_seq1	glucokinase regulator family protein	0	XP_753115	A. fumigatus
comp5642_c0_seq7	glucokinase regulator family protein	0	XP_753115	A. fumigatus
comp3939_c0_seq3	rho guanine nucleotide exchange factor scd1	0	XP_002911728	C. cinerea

comp1847_c0_seq3	tyrosinase	6.00E-13	XP_001885552	L. bicolor
