# Supplementary material for: Transcriptome Analysis and Its Application in Identifying Genes Associated with Fruiting Body Development in Basidiomycete Hypsizygus marmoreus
Source: PLoS One. 2015 Apr 2;10(4):e0123025. doi: 10.1371/journal.pone.0123025 (PMC4383556; doi:10.1371/journal.pone.0123025)
Supplement: S5 Table — (PDF) [file pone.0123025.s016.pdf]

S5_Table. The unigenes of *H. marmoreus* putatively involved in the mTOR signaling pathway.

EST ID	Gene name	E-value	Accession no.	Organism	KEGG ID
comp1337_c0_seq1	EIF4E	0	EGN92279	S. lacrymans	-
comp215_c0_seq1	RP-S6e	0	XM_002469127	P. placenta	K02991
comp1541_c0_seq1	EIF4B	0	EGN99014	S.lacrymans	K03258
comp1302_c0_seq1	RHEB	0	XP_001879802	L. bicolor	K07208
comp14045_c0_seq1	mTOR	0	XP_001829483	C. cinerea	K07204
comp13536_c0_seq1	FRAPTOR	0	EGN96671	S.lacrymans	K07203
comp4335_c0_seq1	ERK1_2	0	EJF57847	D.qualens	K04371
comp1639_c0_seq1	ERK1_2	0	EGO03785	S. lacrymans	K04371
comp2926_c0_seq1	CPKC	0	XP_001876242	L.bicolor	K02677
comp1821_c0_seq1	GBL	0	EIN07757	P.strigosozonata	K08266
comp2950_c1_seq2	MO25	0	EGO19930	S.lacrymans	K08272
comp5332_c0_seq2	PDPK1	0	EIN11732	P.strigosozonata	K06276
comp6196_c0_seq1	ATG1	0	EIN14702	P.strigosozonata	K08269
comp9766_c0_seq1	RPS6KB	8.0E-26	XP_001769870	P.patens	K04688
comp18380_c0_seq1	S6K1/2	0	XP_001881407	L. bicolor	-
